# Effects of *Spirulina* on the functions and redox status of auditory system in senescence-accelerated prone-8 mice

**DOI:** 10.1371/journal.pone.0178916

**Published:** 2017-06-21

**Authors:** Yin-Ching Chan, Juen-Haur Hwang

**Affiliations:** 1 Department of Food and Nutrition, Providence University, Taichung, Taiwan; 2 Department of Otolaryngology, Dalin Tzu Chi Hospital, Buddhist Tzu Chi Medical Foundation, Chiayi, Taiwan; 3 School of Medicine, Tzu Chi University, Hualien, Taiwan; Universidad Pablo de Olavide, SPAIN

## Abstract

To our knowledge, the effects of *Spirulina platensis* water extract (SP) on hearing function have not yet been reported. This study investigated the effects of SP on the function and redox status of the auditory system. Auditory brainstem responses and redox status were compared between two groups of 3-month-old senescence-accelerated prone-8 (SAMP8) mice: the control group was fed a normal diet, and the experimental group was fed a normal diet with oral supplementation of SP for 6 weeks. Compared with the control group, the experimental group had significantly lower hearing thresholds according to auditory brainstem responses measured using click sounds and 8-kHz tone burst sound stimulation at the end of this study. The experimental group had a shorter I-III interval of auditory brainstem responses with 16-kHz tone burst stimulation than the control group that was of borderline significance. Additionally, the experimental group had significantly higher mRNA expression of the superoxide dismutase and catalase genes in the cochlea and brainstem and significantly higher mRNA expression of the glutathione peroxidase gene in the cochlea. Further, the experimental group had significantly lower malondialdehyde levels in the cochlea and brainstem than the control group. However, tumor necrosis factor–α mRNA expression was not significantly different between the control and experimental groups. SP could decrease hearing degeneration in senescence-accelerated prone-8 mice possibly by increasing superoxide dismutase, catalase, and glutathione peroxidase gene expression and decreasing damage from oxidative stress in the cochlea and brainstem.

## Introduction

Sensorineural hearing loss is the most common sensory disorder in older patients. The central auditory system and peripheral hearing organs deteriorate with age. In general, hearing deteriorates more quickly in the central auditory system than the peripheral hearing organs; more quickly and severely in males than females; and more severely at higher than low frequencies [1–3]. Many etiologies have been associated with age-related hearing impairment (ARHI). For example, genetic susceptibility, obesity, obstructive sleep apnea, hypertension, diabetes, dyslipidemia, noise and chemical exposure, alcohol, tobacco, ototoxic medication, diet, hormonal factors, and socioeconomic status [4–11].

Of all hypotheses, damage secondary to oxidative stress is believed to be the most important underlying mechanism of ARHI in both animal and human studies [12–14]. In mice with ARHI, the glutathione peroxidase 6 (GPx) gene is upregulated, and the thioredoxin reductase 1 gene is downregulated [13]. In humans, plasma reactive oxygen species levels are correlated with ARHI severity [14]. Thus, it is reasonable to expect that improved endogenous antioxidant capacity, exogenous antioxidant supplementation, reduced oxidative stress in the auditory system, or any combination of these factors may protect hearing deterioration. For example, vitamin C, vitamin E, coffee, and caffeine could prevent an animal’s hearing loss, auditory neuropathy, or both [12, 15, 16]. Folic acid supplementation could slow hearing decline at low frequencies in subjects with lower folic acid intake [17].

*Spirulina platensis* is a type of blue-green algae. Previous studies showed that *Spirulina platensis* water extract (SP), including its active ingredient, *C-phycocyanin*, have antioxidative and anti-inflammatory effects. These effects might be because of its capability to inhibit cyclooxygenase-2, nicotinamide adenine dinucleotide phosphate (NADPH) oxidase enzymes, or both [17, 18]. In experimental studies, SP slowed memory loss in mice by decreasing oxidative damage and increasing catalase (CAT) activity in the hippocampus, striatum, and cortex [19]. SP could reduce salicylate-induced tinnitus possibly by downregulating the mRNA and protein expression of N-methyl-D-aspartate receptor 2B, proinflammatory cytokines, and cyclooxygenase-2 genes in the cochlea and inferior colliculus of mice [20]. SP could also decrease the overexpression of the manganese-superoxide dismutase (Mn-SOD) gene, as well as malondialdehyde (MDA) levels, but could increase the expression of downregulated CAT genes in many brain regions in salicylate-induced tinnitus [21].

The effects of SP on ARHI in animals or humans have been unclear. Therefore, we aimed to investigate this issue. We hypothesized that SP could prevent hearing degeneration, modulate antioxidant gene expression, and reduce oxidative stress in senescence-accelerated prone-8 (SAMP8) mice.

## Materials and methods

### Animals

Eleven-month-old male SAMP8 mice (n = 12) were randomly divided into two groups (n = 6 each): the control group was fed a normal diet (Fwusow Industry Co, Ltd, Taiwan), and the SP group was fed a normal diet with SP water extract supplementation for 6 weeks (400 mg/kg body weight).

The SP used in this study was supplied by Far East Bio-tec Co, Ltd (Taipei, Taiwan). In brief, SP was prepared as follows: *Spirulina platensis* powder and pure water were mixed to form a suspension; *Spirulina platensis* cells in suspension were disrupted at a temperature lower than room temperature for 24 hours and centrifuged; and the extract (supernatant) was collected and lyophilized. The lyophilized SP contained 15–25% phycobiliproteins (C-phycocyanin and allophycocyanin), 35–45% polysaccharides, 10–20% proteins other than phycobiliproteins, 5–8% water, and 10–12% ash. The well-known active compounds in the extract were sulfated polysaccharides and phycobiliproteins.

The animals were housed (in groups of four mice per cage) in a temperature-controlled room with a constant 12-hr light–dark cycle. Food and tap water were freely available throughout the experiments. The Institutional Animal Care and Use Committee of Dalin Tzu Chi Hospital approved the protocol used in this study.

### Auditory brainstem responses

Auditory brainstem responses (ABR) were measured in the mice when they were 11 months of age and at the end of the study (i.e., at 12.5 months of age) under general anesthesia with an intraperitoneal injection of sodium pentobarbital (65 mg/kg). ABRs (Intelligent Hearing Systems, Miami, FL) were measured in a double-walled, soundproof booth. Subdermal needles were used as electrodes for recording. The active electrode was inserted at the vertex; the reference electrode was ventrolateral to the left ear; and the ground electrode to the low back above the tail. Click sounds, which reflected thresholds around 4 kHz, and 8- and 16-kHz tone bursts were delivered sequentially to the left ear through earphones (Telephonics Corp, Farmingdale, NY). The amplified responses were then averaged by a computer and displayed on a computer screen.

**Fig 1:**
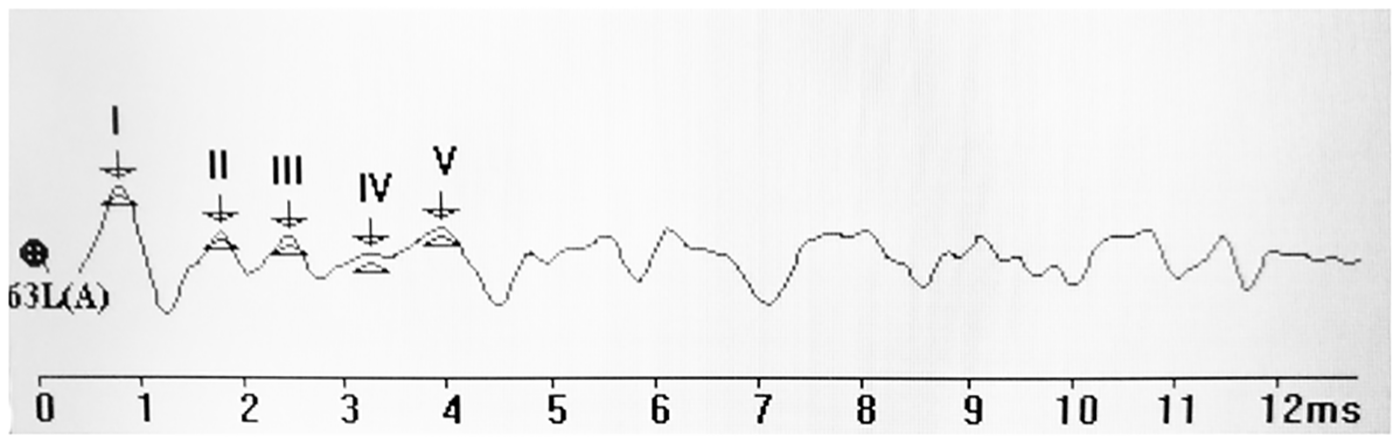
ABR waves for determination of hearing thresholds in mice. The ABR thresholds were determined by the presence of well-defined ABR waves (I-V).

ABR thresholds were obtained for each animal by reducing the stimulus intensity in 5-dB intervals and increasing the stimulus intensity in 3-dB intervals to identify the lowest intensity at which ABR waves I-V were detected by one well-trained audiologist who was blinded to the groups. The respective ABR waves are shown in Fig 1. The ABR data were stored digitally on disks for offline measurements and analysis of latency of ABR components later.

### Isolation of samples and RNA extraction

At the end of the study, the mice were sacrificed by decapitation under general anesthesia with an intraperitoneal injection of pentobarbital (65 mg/kg). The cochlear and brainstem pairs from each animal were immediately dissected using a Zeiss stereomicroscope and stored separately at –80°C until use. RNA isolation was performed using the RNA-bee isolation reagent (Friendswood, USA) with a tissue homogenizer according to the manufacturer’s protocol. The RNA quality was assessed with an Agilent Bioanalyzer 2100, and the ratio of absorbance at 260 and 280 nm was assessed using a nanodrop.

### Reverse transcription–polymerase chain reaction

Reverse transcription–polymerase chain reaction (RT-PCR) was performed separately for each animal. Total RNA was isolated with a PureLink RNA Mini Kit from Ambion RNA by Life Technologies. Approximately one-half of the obtained product was reverse transcribed using a MasterAmp high-fidelity RT-PCR kit from Epicentre (an Illumina company). A PCR reaction was then performed with SOD, CAT, and GPx primers for 35 cycles of denaturation (95°C, 30 seconds), annealing (60°C, 30 seconds), and extension (72°C, 45 seconds). As a control, β-actin was PCR amplified from all samples using the same conditions as with each gene.

### Quantitation of PCR products with Southern blot

The DNA products were measured using Southern blot with a Mini Horizontal Electrophoresis System (MJ-105/MP-100, Major Science, Taiwan) and an E-box-1000/26M inspection certificate and analysis system (E-box Spp-010 E-capt software, USA). The expression levels for all target genes are presented as a relative ratio in comparison to β-actin.

### Measurement of MDA activity

Levels of peroxidized lipids were indirectly determined by measuring the levels of MDA (a by-product of lipid peroxidation) with a commercial kit (BioVision Inc). Tissue samples were homogenized on ice in an MDA lysis buffer (with 1× butylated hydroxytoluene). The MDA level was measured by quantifying the absorbance at 532 nm (nmol). MDA levels were determined by assessing a standard MDA curve. Absorbance values were measured using an Anthos Zenyth 3100 microplate multimode detector (Anthos Labtec Instruments, Austria).

### Statistical analysis

All data are expressed as mean ± standard deviation (S.D.) unless indicated otherwise. ABR thresholds; I-III, I-V, and III-V wave intervals; target gene mRNA expression; and MDA level were compared between the two groups using a Student’s *t* test with Welch’s approximation. All analyses were performed using STATA 10.0 software (Stata Corp, LP, College Station, TX). *P* values of <0.05 were considered significant. The original data of this experiment was shown in S1 File.

## Results

Table 1 shows the ABR thresholds for both groups at the beginning and end of this study. The ABR thresholds were not significantly different between the control and SP groups in clicks (66.2 ± 9.2 versus 68.2 ± 6.4 dB SPL, respectively; *P* = 0.6713), 8-kHz tone burst stimulation (49.8 ± 13.1 versus 52.8 ± 9.3 dB SPL, respectively; *P* = 0.6565), or 16-kHz tone burst stimulation (64.5 ± 7.6 versus 66.8 ± 5.3 dB SPL, respectively; *P* = 0.5518) at the beginning of this study. However, the ABR thresholds were significantly different between the control and SP groups with 8-kHz tone burst stimulation (66.5 ± 12.1 versus 53.0 ± 4.0 dB SPL, respectively; *P* = 0.0382) but were not significantly different with click sound stimulation (81.2 ± 12.6 versus 69.5 ± 3.7 dB SPL, respectively; *P* = 0.0709) or 16-kHz tone burst stimulation (74.3 ± 12.0 versus 68.3 ± 3.3 dB SPL, respectively; *P* = 0.2804) at the end of this study.

**Table 1:** ABR thresholds of the both groups at the beginning and the end of this study.

Mean ± SD (dB SPL)	Control group	SP group	95% CI of difference*	p values**
At the beginning				
Click sound	66.2±9.2	68.2±6.4	-12.1~8.1	0.6713
8 kHz tone burst	49.8±13.1	52.8±9.3	-17.5~11.5	0.6565
16 kHz tone burst	64.5±7.6	66.8±5.3	-10.7~6.1	0.5518
At the end				
Click sound	81.2±12.6	69.5±3.7	-1.3~24.7	0.0709
8 kHz tone burst	66.5±12.1	53.0±4.0	1.0~26.0	0.0382
16 kHz tone burst	74.3±12.0	68.3±3.3	-6.4~18.4	0.2804

Abbreviations: ABR: auditory brainstem response; CI: confident interval; dB SPL: decibel sound pressure level; SD: standard deviation.

*Value of control group minus SP group.

**Student’s *t* test with Welch’s approximation.

Table 2 shows the ABR intervals for both groups with click sound tests at the beginning and end of this study. We measured differences in ABRs between the control and SP groups and demonstrated that the I-III (1.2 ± 0.2 versus 1.2 ± 0.1 ms, respectively; *P* = 0.6150), I-V (3.2 ± 0.3 versus 3.2 ± 0.2 ms, respectively; *P* = 0.9732), and III-V (2.0 ± 0.3 versus 2.0 ± 0.2 ms, respectively; *P* = 0.7935) intervals with click sound stimulation were not significantly different at the beginning of this study. Further, as for differences between in the control and SP groups at the end of the study, the I-III (1.3 ± 0.1 versus 1.1 ± 0.3 ms, respectively; *P* = 0.2373), I-V (3.4 ± 0.5 versus 3.2 ± 0.2 ms, respectively; *P* = 0.4213), and III-V (2.1 ± 0.4 versus 2.1 ± 0.2 ms, respectively; *P* = 0.9273) intervals of ABRs with click sound stimulation were not significantly different.

**Table 2:** ABR intervals by click sound of the both groups at the beginning and the end of this study.

Mean ± SD	Control group	SP group	95% CI of difference*	p values**
At the beginning				
I-III	1.2±0.2	1.2±0.1	-0.1~0.2	0.6150
I-V	3.2±0.3	3.2±0.2	-0.3~0.3	0.9732
III-V	2.0±0.3	2.0±0.2	-0.3~0.3	0.7935
At the end				
I-III	1.3±0.1	1.1±0.3	-0.1~0.5	0.2373
I-V	3.4±0.5	3.2±0.2	-0.3~0.7	0.4213
III-V	2.1±0.4	2.1±0.2	-0.4~0.5	0.9273

Abbreviations: ABR: auditory brainstem response; CI: confident interval; SD: standard deviation.

*Value of control group minus SP group. And, unit in the each cell was msec.

** Student’s t-test with Welch’s approximation.

Table 3 shows the ABR intervals for both groups with 8-kHz tone burst sound at the beginning and end of this study. For the control and SP groups, the I-III (1.2 ± 0.3 versus 1.4 ± 0.3 ms, respectively; *P* = 0.3044), I-V (3.0 ± 0.3 versus 3.1 ± 0.2 ms, respectively; *P* = 0.5029), and III-V (1.8 ± 0.4 versus 1.7 ± 0.2 ms, respectively; *P* = 0.7266) intervals of ABRs with 8-kHz tone burst stimulation were not significantly different at the beginning of this study. At the end of the study, the I-III (1.6 ± 0.7 versus 1.4 ± 0.4 ms, respectively; *P* = 0.4696), I-V (3.6 ± 0.7 versus 3.0 ± 0.2 ms, respectively; *P* = 0.1037), and III-V (2.0 ± 0.3 versus 1.7 ± 0.4 ms, respectively; *P* = 0.1462) intervals of ABR with 8-kHz tone burst stimulation were not significantly different between the control and SP groups.

**Table 3:** ABR intervals by 8k tone burst sound of the both groups at the beginning and the end of this study.

Mean ± SD	Control group	SP group	95% CI of difference*	p values**
At the beginning				
I-III	1.2±0.3	1.4±0.3	-0.5~0.2	0.3044
I-V	3.0±0.3	3.1±0.2	-0.4~0.2	0.5029
III-V	1.8±0.4	1.7±0.2	-0.4~0.5	0.7266
At the end				
I-III	1.6±0.7	1.4±0.4	-0.5~1.0	0.4696
I-V	3.6±0.7	3.0±0.2	-0.2~1.3	0.1037
III-V	2.0±0.3	1.7±0.4	-0.1~0.8	0.1462

Abbreviations: ABR: auditory brainstem response; CI: confident interval; SD: standard deviation.

*Value of control group minus SP group. And, unit in the each cell was msec.

** Student’s t-test with Welch’s approximation.

Table 4 shows the ABR intervals for both groups with 16-kHz tone burst sound at the beginning and end of this study. The I-III (1.2 ± 0.3 versus 1.4 ± 0.2 ms, respectively; *P* = 0.1109), I-V (3.2 ± 0.3 versus 3.1 ± 0.3 ms, respectively; *P* = 0.5988), and III-V (2.0 ± 0.4 versus 1.7 ± 0.3 ms, respectively; *P* = 0.1071) intervals of ABR with 16-kHz tone burst stimulation were not significantly different between the control and SP groups at the beginning of this study. However, the I-III interval (1.7 ± 0.5 versus 1.2 ± 0.3 ms, respectively; *P* = 0.0461), but not the I-V (3.4 ± 0.5 versus 3.0 ± 0.2 ms, respectively; *P* = 0.1149) or III-V (1.7 ± 0.3 versus 1.8 ± 0.4 ms, respectively; *P* = 0.6079) interval, of ABR with 16-kHz tone burst stimulation was significantly different between the control and SP groups at the end of this study.

**Table 4:** ABR intervals by 16k tone burst sound of the both groups at the beginning and the end of this study.

Mean ± SD	Control group	SP group	95% CI of difference*	p values**
At the beginning				
I-III	1.2±0.3	1.4±0.2	-0.6~0.1	0.1109
I-V	3.2±0.3	3.1±0.3	-0.3~0.5	0.5988
III-V	2.0±0.4	1.7±0.3	-0.1~0.8	0.1071
At the end				
I-III	1.7±0.5	1.2±0.3	0.01~1.0	0.0461
I-V	3.4±0.5	3.0±0.2	-0.1~1.0	0.1149
III-V	1.7±0.3	1.8±0.4	-0.6~0.4	0.6079

Abbreviations: ABR: auditory brainstem response; CI: confident interval; SD: standard deviation.

*Value of control group minus SP group. And, unit in the each cell was msec.

** Student’s t-test with Welch’s approximation.

**Fig 2:**
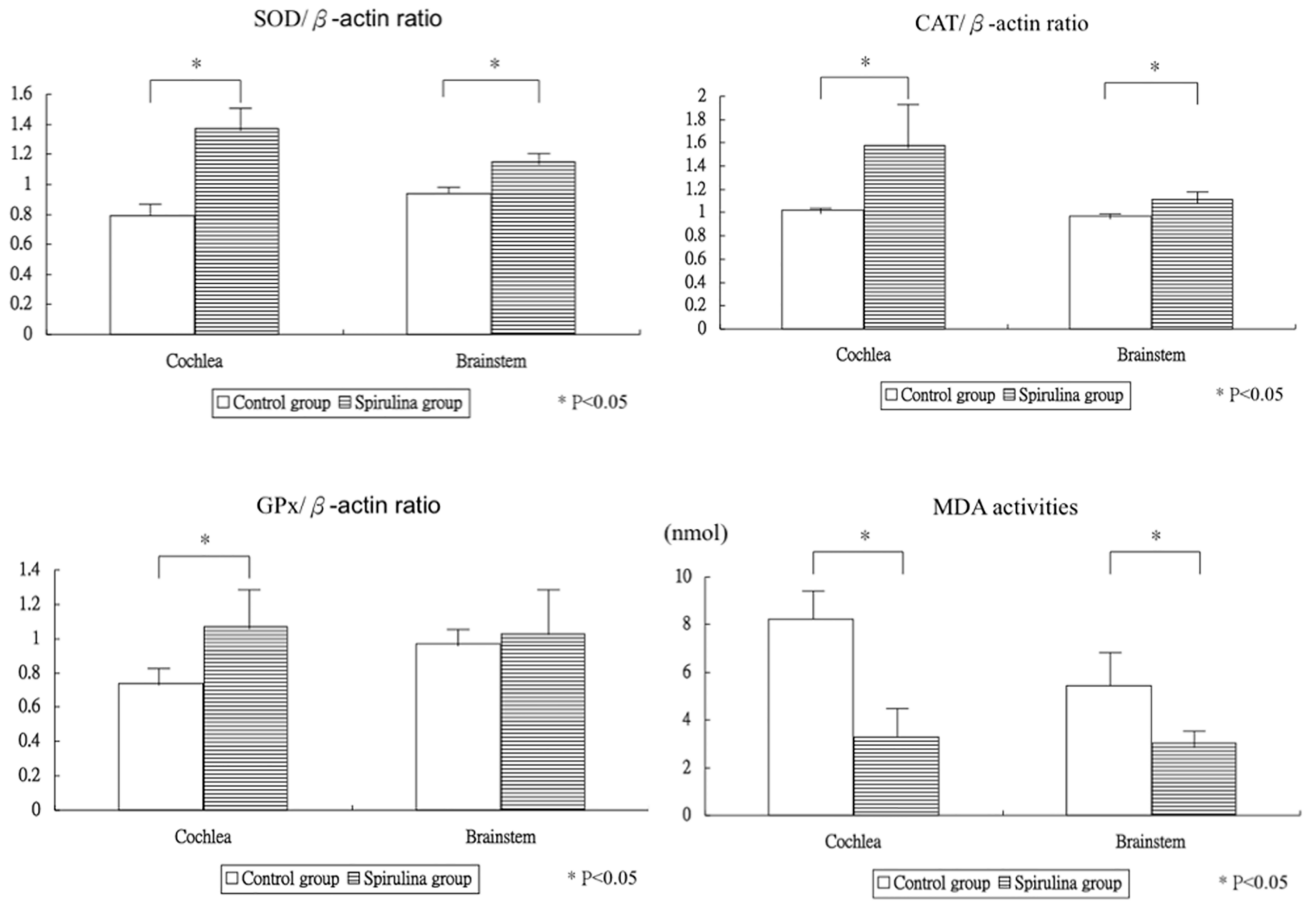
mRNA expression of antioxidant and MDA levels at the end of this study. (a) The SP group had significantly higher expression of the SOD gene in the cochlea and brainstem than the control group. (b) The SP group had higher expression of the CAT gene in the cochlea and brainstem than the control group. (c) Compared with the control group, the SP group had higher expression of the GPx gene in the cochlea, but not in the brainstem. (d) The SP group had significantly lower MDA activity in the cochlea and brainstem.

Compared with the control group, the SP group had a significantly higher mRNA expression of the SOD gene in the cochlea (0.8 ± 0.1 versus 1.4 ± 0.2, respectively; 95% confidence interval [CI] for the control/SP difference = -0.8 to -0.4; *P* = 0.0002) and brainstem (0.9 ± 0.05 versus 1.1 ± 0.06, respectively; 95% CI control/SP difference = -0.3 to -0.1, respectively; *P* < 0.0001) (Fig 2a). Second, the SP group had a significantly higher mRNA expression of the CAT gene in the cochlea (1.0 ± 0.03 versus 1.6 ± 0.36, respectively; 95% CI control/SP difference = -0.9 to -0.2; *P* = 0.0125) and brainstem (1.0 ± 0.04 versus 1.1 ± 0.05, respectively; 95% CI control/SP difference = -0.2 to -0.1, respectively; *P* = 0.0003) (Fig 2b). Third, as compared with the control group, the SP group had significantly higher mRNA expression of the GPx gene in the cochlea (0.7 ± 0.09 versus 1.1 ± 0.20, 95% CI control/SP difference = -0.5 to -0.1; *P* = 0.0054), but not in the brainstem (1.0 ± 0.13 versus 1.0 ± 0.32, respectively; 95% CI control/SP difference = -0.40 to 0.3; *P* = 0.7014) (Fig 2c). However, the SP group did not have a significantly lower expression of the TNF-α gene in the cochlea (0.7 ± 0.04 versus 0.8 ± 0.11, respectively; 95% CI control/SP difference = -0.1 to 0.1, = 0.6907) or brainstem (1.5 ± 0.32 versus 1.3 ± 0.37, respectively; 95% CI control/SP difference = -1.2 to 1.5; *P* = 0.7675). However, the SP group had significantly lower MDA activity in the cochlea (8.2 ± 1.16 versus 3.3 ± 1.37, respectively; 95% CI control/SP difference = 3.3 to 6.5; *P* < 0.0001) and brainstem (5.4 ± 1.45 versus 3.0 ± 0.56, respectively; 95% CI control/SP difference = 0.9 to 3.9; *P* = 0.0067) (Fig 2d).

## Discussion

This study demonstrated that SP diet supplementation could slow the deterioration of hearing thresholds with click sound and 8-kHz tone burst stimulation in SAMP8 mice. However, SP decreased only the I-III interval of ABRs with 16-kHz tone burst stimulation with borderline significance. SP could increase mRNA expression of the SOD and CAT genes but decrease MDA concentration in the cochlea and brainstem. It increased mRNA expression of the GPx gene only in the cochlea, but not in the brainstem.

These differential findings may raise an issue regarding ABR changes with aging and their responses to exogenous antioxidant supplementation. First, previous studies have shown that age, gender, hearing threshold, stimulation intensity, and side have significant influences on ABR latencies, interwave intervals, or both [22,23]. Older patients have increased latencies and interwave intervals than younger patients [24]. SAMP1 and senescence-accelerated resistant mice (SAMR1) mice have age-related auditory loss expressed as elevated thresholds and prolonged I-III and I-IV intervals, especially at high frequencies [25]. Age-related threshold shifts and increased amplitude reductions were observed, but no changes were demonstrated in latencies or interwave intervals in guinea pigs [26]. Females have shorter latencies, shorter I-V or III-V intervals, and higher amplitudes than males [27]. Hearing loss was related to wave V latency, but not with waves I or III [28]. However, Watson [29] demonstrated that both wave I and V had latency prolongation with increasing levels of hearing loss. Furthermore, subjects had larger wave V amplitudes and shorter interwave intervals elicited from the right ear than the left ear [30]. The interaural differences in III-V intervals could be negatively correlated with hearing asymmetry [31]. According to the findings on the effects of aging on ABRs, threshold shifts and amplitude reductions might occur earlier than the prolongation of latencies or interwave intervals, as shown in a report by Proctor [26].

Second, some studies have demonstrated the effects of exogenous antioxidant supplementation on various components of ABRs. For example, lecithin could help preserve cochlear mitochondrial function and lessen age-related ABR threshold elevations in rats [32]. Additionally, vitamins B, E, or C or L-carnitine reduced cisplatin-induced ABR threshold elevation and interwave I-IV interval prolongation in rats [33]. Resveratrol might attenuate cisplatin-induced ototoxicity, as shown by hearing threshold values, wave I and IV latencies, and I-IV intervals [34]. Following antioxidant administration, increased ABR interwave intervals were restored in a neonatal rat model of hypoxic ischemic brain injury [35]. In humans, women who were treated with hormone replacement therapy had shorter wave latencies and interwave latencies than postmenopausal women in the control group [36]. In this study, SP slowed the deterioration of ABR thresholds, but not interwave intervals. Thus, we think exogenous antioxidant supplementation might have greater protective effects on ABR threshold shifts than on ABR interwave interval prolongation.

The SAMP8 strain experiences premature hearing loss and cochlear degeneration. The mechanisms underlying premature hearing loss in SAMP8 mice involves oxidative stress; altered antioxidant enzymes levels; and decreased complex I, II, and IV activity, which in turn leads to chronic inflammation and triggers the apoptotic pathway, autophagic cell death pathway, or both [37]. As for redox status, SOD activity was lower at 1 month in SAMP8 mice than in SAMR1 mice that had further declined in both strains at 9 months. No significant difference was found in cochlear CAT activity between 1-month-old SAMR1 mice and 1-month-old SAMP8 mice. At 9 months, although CAT activity had been maintained in SAMR1 mice, CAT activity was greatly reduced in SAMP8 mice [36]. MDA levels were significantly higher in the cochlea of SAMP8 mice than in those of SAMR1 mice. In this study, we demonstrated that SP could increase SOD, CAT, and GPx gene expression and decrease MDA levels in the cochlea and brainstem of 9-month-old SAMP8 mice.

Previous studies determined that the first stage of activation in the redox system occurs at the plasma membrane. NADPH oxidase localized in the plasma membrane reduces oxygen to superoxide anion radicals, which are then dismuted to hydrogen peroxide and oxygen by SOD. Intracellular enzymes including CAT, GPx, and heme oxygenase (HO)-1 then catalyze the breakdown of hydrogen peroxide (H_2_O_2_); H_2_O_2_ is also converted to hypochlorous acid (HOCl) by myeloperoxidase. Moreover, SP and its active ingredient have antioxidative and anti-inflammatory effects through the inhibition of NADPH oxidase enzymes [18]. Thus, it is reasonable to determine if SP could modulate gene expression, enzymatic activity, or both regarding SOD, CAT, GPx, HO-1, or any combination of these. This hypothesis regarding the impact of SP on redox status has been reported in some previous studies. For example, SP reduced oxidative damage and augmented CAT activity in the hippocampus, striatum, and cortex of SAMP8 mice [19]. SP decreased salicylate-induced overexpression of Mn-SOD genes and MDA levels but increased salicylate-induced downregulation of CAT genes in many brain regions [21]. SP downregulated TNF-α mRNA and protein expression in the cochlea and inferior colliculus of SAMP8 mice with salicylate-induced tinnitus [20].

As shown in different studies on antioxidants, SP might have a differential impact or even the opposite effect on gene expression, enzymatic activities, or both. SP might augment antioxidant expression, enzymatic activities, or both in SAMP8 mice during normal aging as shown in a memory loss study [19] and in this ARHI study. However, SP might have the opposite effect on antioxidant gene expression, enzymatic activities, or both in SAMP8 mice with salicylate-induced tinnitus [20, 21]. Similarly, unlike the findings in another study on salicylate-induced tinnitus (20), SP did not alter the gene expression of TNF-α in the cochlea or brainstem in SAMP8 mice during normal aging in this study.

## Conclusions

SP diet supplementation could slow hearing threshold deterioration and decrease the I-III interval of ABRs with 16-kHz tone burst stimulation in SAMP8 mice. The beneficial effects of SP on auditory functions were possibly associated with increasing mRNA expression of SOD, CAT, and GPx genes and decreasing MDA concentration in the cochlea and brainstem.

## Supporting information

S1 File The original data of this experiment was shown. (XLS)

## References

[1] Enrietto JA, Jacobson KM, Baloh RW. Aging effects on auditory and vestibular responses: a longitudinal study. Am J Otolaryngol 1999; 20(6): 371–378. 10609481 10.1016/s0196-0709(99)90076-5

[2] Snell KB, Frisina DR. Relationships among age-related differences in gap detection and word recognition. J Acoust Soc Am 2000; 107(3): 1615–26. 10738815 10.1121/1.428446

[3] Hwang JH, Li CW, Wu CW, Chen JH, Liu TC. Aging effects on the activation of the auditory cortex during binaural speech listening in white noise: an fMRI study. Audiol Neurotol 2007; 12(5): 285–94. 10.1159/000103209 17536197

[4] Hwang JH, Wu CC, Hsu CJ, Liu TC, Yang WS. Association of central obesity with the severity and audiometric configurations of age-related hearing impairment. Obesity 2009; 17: 1796–801. doi: 10.1038/oby.2009.66 19300432 10.1038/oby.2009.66

[5] Hwang JH, Hsu CJ, Liu TC, Yang WS. Association of plasma adiponectin levels with hearing thresholds in adults. Clin Endocrinol (Oxf). 2011; 75: 614–620. 21535075 10.1111/j.1365-2265.2011.04090.x

[6] Hwang JH, Chen JC, Hsu CJ, Liu TC. Association of obstructive sleep apnea and auditory dysfunctions in older subjects. Otolaryngol Head Neck Surg 2011; 144: 114–119. doi: 10.1177/0194599810390859 21493399 10.1177/0194599810390859

[7] Hwang JH, Hsu CJ, Yu WH, Liu TC, Yang WS. Diet-induced obesity exacerbates auditory degeneration via hypoxia, inflammation, and apoptosis signaling pathways in CD/1 mice. PLOS ONE 2013; 8(4): e60730 doi: 10.1371/journal.pone.0060730 23637762 10.1371/journal.pone.0060730 PMC3637206

[8] Hwang JH, Chen JC, Yang WS, Liu TC. Waist circumference is associated with pitch pattern sequence score in older male adults. Int J Audiol. 2012; 51 (12): 920–925. doi: 10.3109/14992027.2012.721933 23072653 10.3109/14992027.2012.721933

[9] Hwang JH, Tseng FY, Liu TC, Yang WS. No association between plasma adiponectin levels and central auditory function in adults. Metabolic Brain disease 2014; 30: 191–196. doi: 10.1007/s11011-014-9597-1 25108594 10.1007/s11011-014-9597-1

[10] Wu CC, Tsai CH, Lu YC, Lin HC, Hwang JH, Lin YH, et al Contribution of adiponectin and its type 1 receptor to age-related hearing impairment. Neurobiology of Aging 2015; 36: 2085–2093. doi: 10.1016/j.neurobiolaging.2015.02.030 25911279 10.1016/j.neurobiolaging.2015.02.030

[11] Van Eyken, Van Camp G, Van Laer L. The complexity of age-related hearing impairment: contributing environmental and genetic factors. Audiol Neurootol 2007; 12: 345–358. doi: 10.1159/000106478 17664866 10.1159/000106478

[12] Seidman MD, Ahmad N, Bai U. Molecular mechanisms of age-related hearing loss. Ageing Res Rev 2002; 1: 331–343. 12067590 10.1016/s1568-1637(02)00004-1

[13] Tadros SF, D'Souza M, Zhu X, Frisina RD. Age-related gene expression changes for antioxidants in the CBA mouse cochlea. Plos One 2014; 9: e9027919. 10.1371/journal.pone.0090279 PMC3938674 24587312

[14] Hwang JH, Chen JC, Hsu CJ, Yang WS, Liu TC. Plasma reactive oxygen species levels were correlated with severity of age-related hearing impairment in humans. Neurobiol Aging. 2012; 33(9): 1920–1926. doi: 10.1016/j.neurobiolaging.2011.10.012 22133279 10.1016/j.neurobiolaging.2011.10.012

[15] Hong BN, Yi TH, Kim SY, Kang TH. High-dosage pyridoxine-induced auditory neuropathy and protection with coffee in mice. Biol Pharm Bull 2009; 32(4): 597–603. 19336890 10.1248/bpb.32.597

[16] de Rivera C, Shukitt-Hale B, Joseph JA, Mendelson JR. The effects of antioxidants in the senescent auditory cortex. Neurobiol Aging 2006; 27(7): 1035–1044. doi: 10.1016/j.neurobiolaging.2005.05.003 15950320 10.1016/j.neurobiolaging.2005.05.003

[17] Romay C, Ledón N, González R. Phycocyanin extract reduces leukotriene B4 levels in arachidonic acid-induced mouse-ear inflammation test. J Pharm Pharmacol 1999; 51: 641–642. 10411225 10.1211/0022357991772646

[18] McCarty MF, Barroso-Aranda J, Contreras F. Oral phycocyanobilin may diminish the pathogenicity of activated brain microglia in neurodegenerative disorders. Med Hypotheses 2010; 74: 601605. 10.1016/j.mehy.2008.09.061 19576698

[19] Hwang JH, Lee IT, Jeng KC, Wang MF, Hou RC, Wu SM, et al Spirulina prevents memory dysfunction, reduces oxidative stress damage and augments antioxidant activity in senescence-accelerated mice. J Nutr Sci Vitaminol (Tokyo) 2011; 57: 186–191. 21697639 10.3177/jnsv.57.186

[20] Hwang JH, Chen JC, Chan YC. Effects of C-phycocyanin and Spirulina on salicylate-induced tinnitus, expression of NMDA receptor and inflammatory genes. PLoS One 2013; 8(3): e58215 doi: 10.1371/journal.pone.0058215 23533584 10.1371/journal.pone.0058215 PMC3606192

[21] Hwang JH, Chang NC, Chen JC, Chan YC. Expression of antioxidant genes in the mouse cochlea and brain in salicylate-induced tinnitus and effect of treatment with Spirulina platensis water extract. Audiol Neurotol 2015; 20: 322–329. 10.1159/000381934 26277928

[22] Collet L, Berger-Vachon C, Desreux V, Morgon A. Auditory brainstem response (ABR) latency: relative importance of age, sex and sensorineural hearing-loss using a mathematical model of the audiogram. Int J Neu- rosci 1992; 67: 187–197. 10.3109/00207459208994784 1305633

[23] Lightfoot GR. Correcting for factors affecting ABR wave V latency. Br J Audiol 1993; 27: 211–220. 8241970 10.3109/03005369309076695

[24] Oku T, Hasegewa M. The influence of aging on auditory brainstem response and electrocochleography in the elderly. ORL J Otorhinolaryngol Relat Spec 1997; 59(3): 141–146. 9186968 10.1159/000276927

[25] Saitoh Y, Hosokawa M, Shimada A, Watanabe Y, Yasuda N, Takeda T, et al Age-related hearing impairment in senescence-accelerated mouse (SAM). Hear Res. 1994; 75(1–2): 27–37. 8071152 10.1016/0378-5955(94)90052-3

[26] Proctor TB, Velde TM, Dayal VS, Bhattacharyya TK, Artwohl J, Towle VL. Auditory brain stem response in young and old guinea pigs. Am J Otol. 1998; 19(2): 226–229. 9520061

[27] Hultcrantz M, Simonoska R, Stenberg AE. Estrogen and hearing: a summary of recent investigations. Acta Otolaryngol 2006; 126: 10–14. doi: 10.1080/00016480510038617 16308248 10.1080/00016480510038617

[28] Jerger J, Johnson K. Interactions of age, gender, and sensorineural hearing loss on ABR latency. Ear Hear 1988; 9: 168–176. 3169397 10.1097/00003446-198808000-00002

[29] Watson DR. The effects of cochlear hearing loss, age and sex on the auditory brainstem response. Audiology 1996; 35: 246–258. 8937657 10.3109/00206099609071945

[30] Sininger YS, Cone-Wesson B, Abdala C. Gender distinctions and lateral asymmetry in the low-level auditory brainstem response of the human neonate. Hear Res 1998; 126: 58–66. 9872134 10.1016/s0378-5955(98)00152-x

[31] Hwang JH, Chao JC, Ho HC, Hsiao SH. Effects of sex, age and hearing asymmetry on the interaural differences of auditory brainstem responses. Audiol Neurotol 2008; 13: 29–33. 10.1159/000107468 17715467

[32] Seidman MD, Khan MJ, Tang WX, Quirk WS. Influence of lecithin on mitochondrial DNA and age-related hearing loss. Otolaryngol Head Neck Surg. 2002; 127(3): 138–144. doi: 10.1067/mhn.2002.127627 12297801 10.1067/mhn.2002.127627

[33] Tokgöz SA, Vuralkan E, Sonbay ND, Çalişkan M, Saka C, Beşalti Ö, et al Protective effects of vitamins E, B and C and L-carnitine in the prevention of cisplatin-induced ototoxicity in rats. J Laryngol Otol. 2012; 126(5): 464–469. doi: 10.1017/S0022215112000382 22490890 10.1017/S0022215112000382

[34] Simşek G, Tokgoz SA, Vuralkan E, Caliskan M, Besalti O, Akin I. Protective effects of resveratrol on cisplatin-dependent inner-ear damage in rats. Eur Arch Otorhinolaryngol. 2013; 270(6): 1789–1793. doi: 10.1007/s00405-012-2183-4 23001434 10.1007/s00405-012-2183-4

[35] Revuelta M, Arteaga O, Montalvo H, Alvarez A, Hilario E, Martinez-Ibargüen A. Antioxidant treatments recover the alteration of auditory-evoked potentials and reduce morphological damage in the inferior colliculus after perinatal asphyxia in rat. Brain Pathol. 2016; 26(2): 186–98. doi: 10.1111/bpa.12272 25990815 10.1111/bpa.12272 PMC8029321

[36] Caruso S, Cianci A, Grasso D, Agnello C, Galvani F, Maiolino L, et al Auditory brainstem response in postmenopausal women treated with hormone replacement therapy: a pilot study. Menopause. 2000; 7(3): 178–183. 10810963 10.1097/00042192-200007030-00008

[37] Menardo J, Tang Y, Ladrech S, Lenoir M, Casas F, Michel C, et al Oxidative stress, inflammation, and autophagic stress as the key mechanisms of premature age-related hearing loss in SAMP8 mouse cochlea. Antioxid. Redox Signal. 2012; 16, 263–274. doi: 10.1089/ars.2011.4037 21923553 10.1089/ars.2011.4037

