# Correction: Effects of *Spirulina* on the functions and redox status of auditory system in senescence-accelerated prone-8 mice

**DOI:** 10.1371/journal.pone.0191349

**Published:** 2018-01-11

**Authors:** Yin-Ching Chan, Juen-Haur Hwang

There is an error in the third sentence in the Abstract. The correct sentence is as follows: Auditory brainstem responses and redox status were compared between two groups of 11-month-old senescence-accelerated prone-8 (SAMP8) mice: the control group was fed a normal diet, and the experimental group was fed a normal diet with oral supplementation of SP for 6 weeks.
